# Emergence of a Fatal ST11-KL64 Tigecycline-Resistant Hypervirulent Klebsiella pneumoniae Clone Cocarrying *bla*_NDM_ and *bla*_KPC_ in Plasmids

**DOI:** 10.1128/spectrum.02539-22

**Published:** 2022-10-07

**Authors:** Jinzhu Huang, Miao Yi, Yaling Yuan, Peiwen Xia, Bingxue Yang, Jiajia Liao, Zijun Dang, Shengli Luo, Yun Xia

**Affiliations:** a Department of Laboratory Medicine, The First Affiliated Hospital of Chongqing Medical University, Chongqing, China; Brown University

**Keywords:** KPC and NDM, ST11-KL64, hvKP, tigecycline resistance

## Abstract

The combination of hypervirulent Klebsiella pneumoniae (hvKP) infection with carbapenem and tigecycline resistance leads to significant challenges to clinical treatment, with limited available antibiotics and poor patient prognoses. The hvKP12 isolate was obtained from a blood sample of a 74-year-old female in a Chinese teaching hospital. Whole-genome sequencing and microbial characterization were performed to understand the evolutionary mechanism of its resistance. The patient infected with hvKP12 died due to pyemia after a 17-day tigecycline treatment. The antimicrobial susceptibility test identified that hvKP12 was resistant to tigecycline and carbapenems. Variants of *tet*(A) and the overexpression of efflux pumps related to tigecycline resistance were detected in hvKP12. Conjugation experiments with *bla*_NDM_ and *bla*_KPC_ plasmids failed in the laboratory environment. Additionally, phylogenetic analysis suggested that hvKP12 was a clinical high-risk clone of ST11-KL64. We found that the *bla*_KPC-2_ gene segment was formed by IS*26*-mediated gene cluster translocation. Interestingly, the evolutionary pathway of hvKP12 suggested that the KPC-2-producing carbapenem-resistant K. pneumoniae (KPC-2–CRKP) strain evolved into a KPC-NDM-CRKP strain by acquiring the NDM plasmid. To our knowledge, this is the first report of tigecycline-resistant ST11-KL64 carbapenem-resistant hvKP (CR-hvKP) bacteria coproducing *bla*_KPC_ and *bla*_NDM_, causing a fatal blood infection.

**IMPORTANCE** Infections with CRKP coproducing KPC and NDM currently have limited clinical antibacterial options, and tigecycline is used as the last line of defense for therapy. However, this study found that CR-hvKP infection with tigecycline resistance, which may lead to many bacteria being resistant to most commonly used antibiotics, brought significant challenges to clinical treatment. The clonal propagation of ST11-KL64 CRKP should receive sufficient attention.

## INTRODUCTION

Hypervirulent Klebsiella pneumoniae (hvKP) and carbapenem-resistant K. pneumoniae (CRKP) have evolved into two major types of clinically significant pathogens in China, often linked to significant morbidity and mortality. The hvKP can affect previously healthy patients and cause severe infections such as liver abscesses, meningitis, endophthalmitis, and necrotizing fasciitis (1). Both KPC and NDM can hydrolyze a wide spectrum of β-lactams; KPC can also hydrolyze monobactams but can be inhibited by avibactam. On the contrary, NDM cannot hydrolyze monobactams, but avibactam cannot inhibit it (2). Strains with the coexistence of KPC and NDM are found worldwide, such as in India (3), Pakistan (4), Brazil (5), and China (6), etc. The coproduction of KPC and NDM carbapenems in a single strain should be taken seriously because it is capable of conferring higher-level carbapenem resistance and mediating the spread of carbapenems genes (7). Sequence type 11 (ST11) is one of the most common clones of CRKP observed in China, and it has received much attention recently since an increasing proportion of clinical strains are hypervirulent and antibiotic resistant (8). To date, most reported hvKP strains have belonged to the K1 and K2 capsular serotypes of K. pneumoniae (9). It has been reported that ST11-KL64 has gradually displaced ST11-KL47 as the most prevalent hypervirulent CRKP clone in China, resulting in a higher fatality rate for infected patients (10). Most importantly, the appearance and increasing prevalence of ST11-K64 CRKP strains necessitate urgent control measures.

Tigecycline, the latest member of the tetracycline family to enter clinics, is a medication for severe CRKP infections and should be used only as a last-resort agent (11). However, tigecycline-resistant CRKP isolates (TCRKP isolates) have been found in different countries (12). The tigecycline resistance mechanism is not fully understood (13). The underlying molecular mechanisms of tigecycline resistance are complex; one of the most common mechanisms observed in *Enterobacteria* is the overexpression of the RND efflux pumps AcrAB and OqxAB (14). Mutations in the *acrR*, *ramR*, *rpsJ*, and *oqxR* genes also play a vital role in tigecycline resistance (15). Recent investigations have demonstrated that widespread mutant *tet*(A) genes are problematic since they may transmit higher tigecycline resistance to K. pneumoniae (16). This study reports the emergence of a tigecycline-resistant ST11-KL64 carbapenem-resistant hvKP isolate cocarrying *bla*_KPC_ and *bla*_NDM_ from a blood infection sample in China.

## RESULTS

### Clinical medical records of the hvKP12 stain.

**FIG 1:**
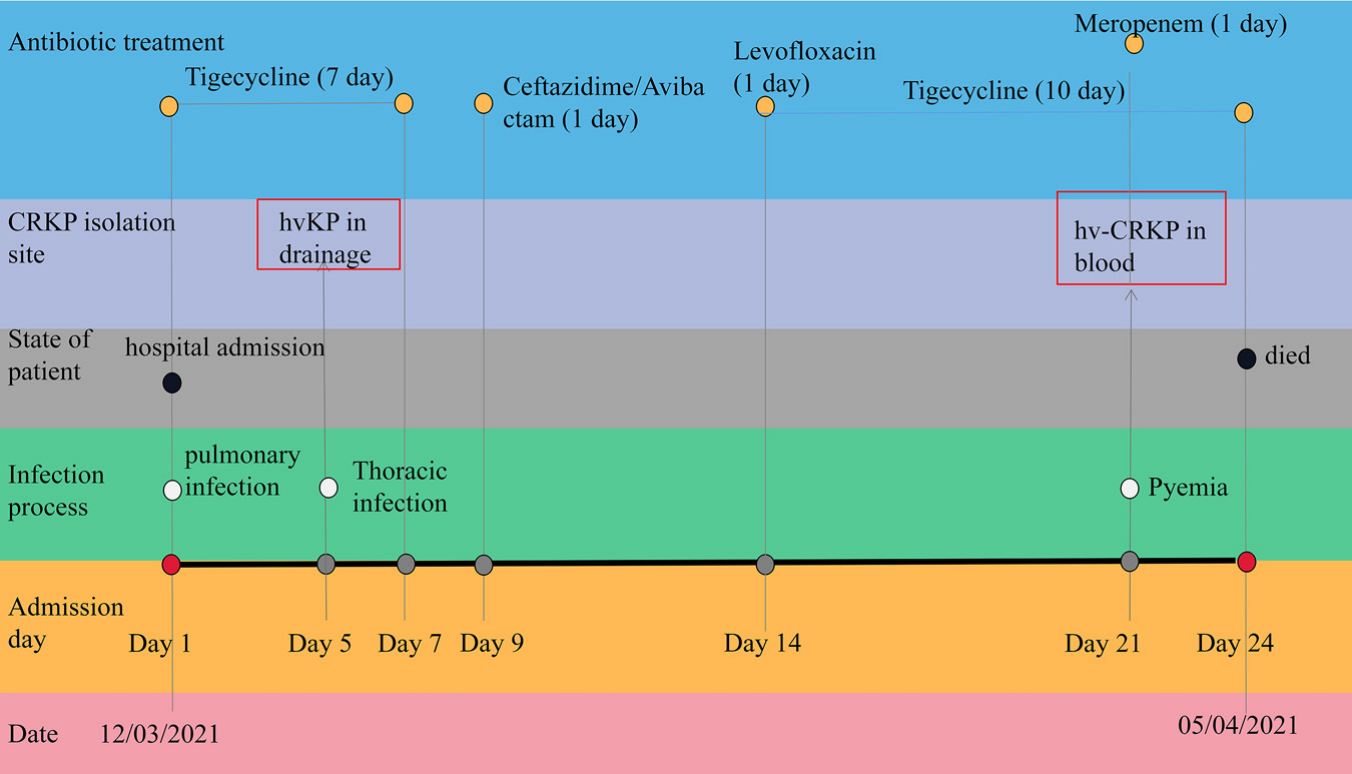
Medical history and antibiotic stewardship of the patient infected with hvKP12.

The carbapenem-resistant *Enterobacteriaceae* (CRE) in our hospital had been constantly monitored and statistically analyzed every year. Medical records documented that hvKP12 was collected from a blood sample of a 72-year-old female in the intensive care unit (ICU) who had basic diseases (hypertension and rheumatoid arthritis). The patient was diagnosed with invasive K. pneumoniae liver abscess syndrome, and then infection lesion kept expanding and eventually developed sepsis. The patient infected with hvKP12 died after 24 days in the hospital. Based on the clinical symptoms and the bacterial culture results, the patient was infected with hypervirulent K. pneumoniae. We supposed that the patient was first infected with hvKP sensitive to almost antibiotics, after a 24-day antibiotic treatment with tigecycline and so on, she died of CR-hvKP causing pyemia (Fig. 1).

### Microbiological characteristics.

Sanger sequencing confirmed that hvKP12 carried the *bla*_KPC_ and *bla*_NDM_ genes. The hvKP12 strain was sensitive to only amikacin, colistin, and aztreonam-avibactam (Table 1). It was resistant to other antibiotics at high levels, except for tigecycline (MIC of 8 mg/L). The string test was negative for hvKP12. Molecular typing classified hvKP12 as belonging to ST11-KL64. According to the conjugation assay, neither the KPC plasmid nor the NDM plasmid could be conjugated for transfer.

**TABLE 1:** Antimicrobial susceptibility results for KPC-NDM-CRE^*a*^

Antimicrobial	hvKP12 MIC (mg/L)
AZT	≥256
CAZ	≥256
IPM	≥256
CRO	≥128
FEP	≥128
MEM	≥128
AMK	**16**
TGC	8
LEV	128
FOX	≥256
CST	**≤** **0.25**
TZP	≥128
SCF	≥256
CAZ-AVI	≥128
AZT-AVI	**1**

a AZT, aztreonam; CAZ, ceftazidime; IMP, imipenem; CRO, ceftriaxone; FEP, cefepime; MEM, meropenem; AMK, amikacin; TGC, tigecycline; LVX, levofloxacin; FOX, cefoxitin; CST, colistin; TZP, piperacillin-tazobactam; SCF, cefoperazone-sulbactam; CAZ-AVI, ceftazidime-avibactam; ATM-AVI, aztreonam-avibactam. Numbers in boldface type indicate susceptibility according to CLSI/EUCAST breakpoints.

### Tigecycline resistance mechanism.

The *tet*(A) variants with type 1 mutations were identified in hvKP12 (16). In hvKP12, there was a new *tet*(A) mutation F163Y. Thus, the nucleotide changes in *ramR* and *oqxR* compared with the reference strain MGH78578 (GenBank accession number CP000647) were A19V and R153C. The *tet*(X) gene was not detected. Moreover, the relative expression levels of *marA* increased 3.01-fold, those of *rarA* increased 2.67-fold, and those of *oqxB* increased 2.30-fold in hvKP12 (Table 2).

**TABLE 2:** Tigecycline resistance genes and qPCR results

Parameter	Value for hvKP12 (TGC MIC, 8 mg/L)
Mutation(s) in gene	
*acrR*	F172S, G164A, F197I, P161R, L195V, R173G, K201M
*ramR*	A19V
*oqxR*	R153C
*rpsJ*	—^*b*^
*tet*(X)	—
*tet*(A)	I5R, V55M, I75V, T84A, F163Y, S201A, F202S, V203F
Mean relative expression level ± SD^*a*^	
*acrA*	0.86 ± 0.12
*acrB*	1.17 ± 0.24
*ramA*	1.18 ± 0.12
*marA*	3.01 ± 1.10
*soxS*	1.63 ± 0.22
*rarA*	2.67 ± 2.08
*oqxB*	2.30 ± 0.18

a Relative expression levels compared with ATCC 25922 (expression = 1). Results are means from 3 runs ± standard deviations.

b —, not determined.

### Genome statistics and gene prediction.

Whole-genome sequencing (WGS) was used to characterize the genome information of hvKP12. The hvKP12 contained a 5,490,858-bp chromosome and eight plasmids, namely, phvKP12-VIR (225,067 bp), phvKP12B (115,543 bp), phvKP12C (87,095 bp), phvKP12-KPC (83,447 bp), phvKP12-NDM (46,161 bp), phvKP12F (11,970 bp), phvKP12G (5,596 bp), and phvKP12H (2,445 bp). Compared with hosts of similar plasmids, phvKP12-NDM and phvKP12H showed high similarity with plasmids from Escherichia coli, and other plasmids were from Klebsiella pneumoniae. The antimicrobial resistance and virulence genes of the hvKP12 isolate were determined using WGS data, and resistance genes are depicted in Table 3. The β-lactam resistance genes included *bla*_KPC-2_, *bla*_NDM-5_, *bla*_SHV-182_, *bla*_SHV-12_, *bla*_CTX-M-65_, and *bla*_LAP-2_. However, other detected resistance genes, such as *qnrS1*, *aadA2b*, *catA2*, *sul1*, *dfrA14*, and *fosA*, had relatively resistance phenotypes including quinolone, aminoglycoside, macrolide, phenicol, sulfonamide, trimethoprim, rifampin, and fosfomycin resistance. In addition to resistance genes, the hvKP12 isolate also possessed virulence genes such as aerobactin (*iucABCD* and *iutA*), hypermucoviscosity (*rmpA* and *rmpA2*), type I fimbria (*fimA–K*), type III fimbria (*mrkA–J*), salmochelin (*iroN* and *iroE*), Ent siderophore (*entA–F* and *fepA–G*), and yersiniabactin (*irp1-irp2* and *ybtA–X*) genes.

**TABLE 3:** Overall features of the hvKP12 genome

Parameter	Value				
	Chromosome	phvKP12-VIR	phvKP12-C	phvKP12-KPC	phvKP12-NDM
Size (bp)	5,490,858	225,067	87,095	83,447	46,161
G+C content (%)	57.33	50.06	53.94	54.97	46.65
No. of predicted ORFs	5,139	231	103	120	43
Resistance gene(s)	*aadA3*, *bla*_SHV-182_, *fosA*, *mdf*(A), *sul1*	None	*bla*_LAP-2_, *catA2*, *qnrS1*, *sul2*, *tet*(A), *dfrA14*	*bla*_CTX-M-65_, *bla*_SHV-12_, *bla*_KPC-2_	*bla*_NDM-5_

### Comparative genomic analysis of hvKP12 plasmids.

**FIG 2:**
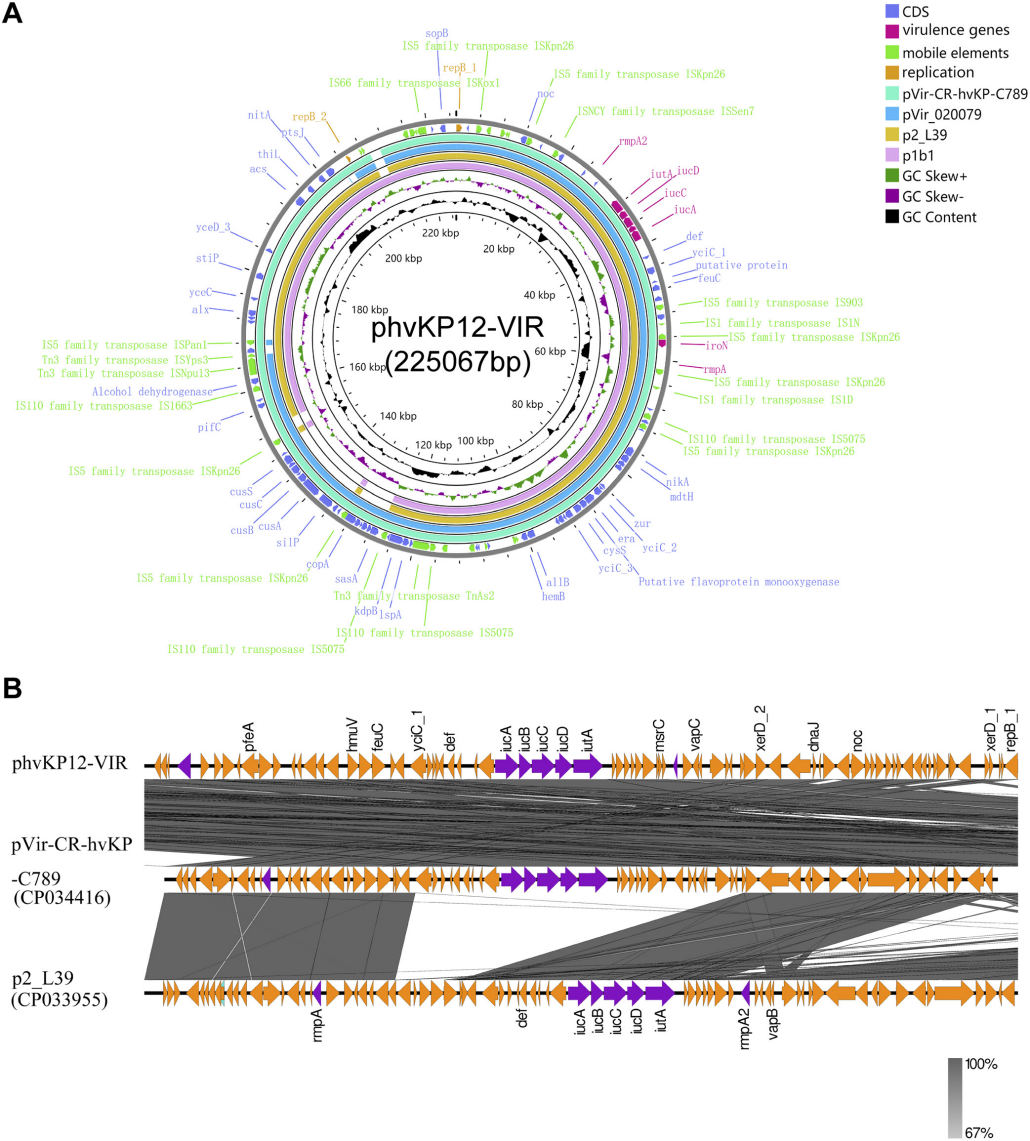
Circular map of the phvKP12-VIR plasmid and comparison with similar plasmids. (A) Circular map of the phvKP12-VIR plasmid and comparison with similar plasmids. CDS, coding DNA sequence. (B) Comparison of virulence genes in the phvKP12-VIR, pVir-CR-hvKP-C789, and p2_L39 plasmids.

The largest plasmid, phvKP12-VIR, belonged to the hybrid IncFIB:IncHI1B type. By BLASTn analysis, the whole plasmid sequence had 99% identity and 95 to 99% query coverage with the plasmids pVir-CR-hvKP-C789 (GenBank accession number CP034416.1), pVir_020079 (accession number CP029383.2), and p2_L39 (accession number CP033955.1) (Fig. 2). The 65-kb virulence region including virulence gens in phvKP12-VIR was highly similar to p2_L39 and pVir-CR-hvKP-C789 (99.9% identity and 99.9% query coverage) (Fig. 2B). The phvKP12-VIR was a typical virulence plasmid with aerobactin genes (*iucABCD* and *iutA*) and hypermucoviscosity genes (*rmpA* and *rmpA2*). In contrast, no antimicrobial resistance gene was discovered in phvKP12-VIR.

**FIG 3:**
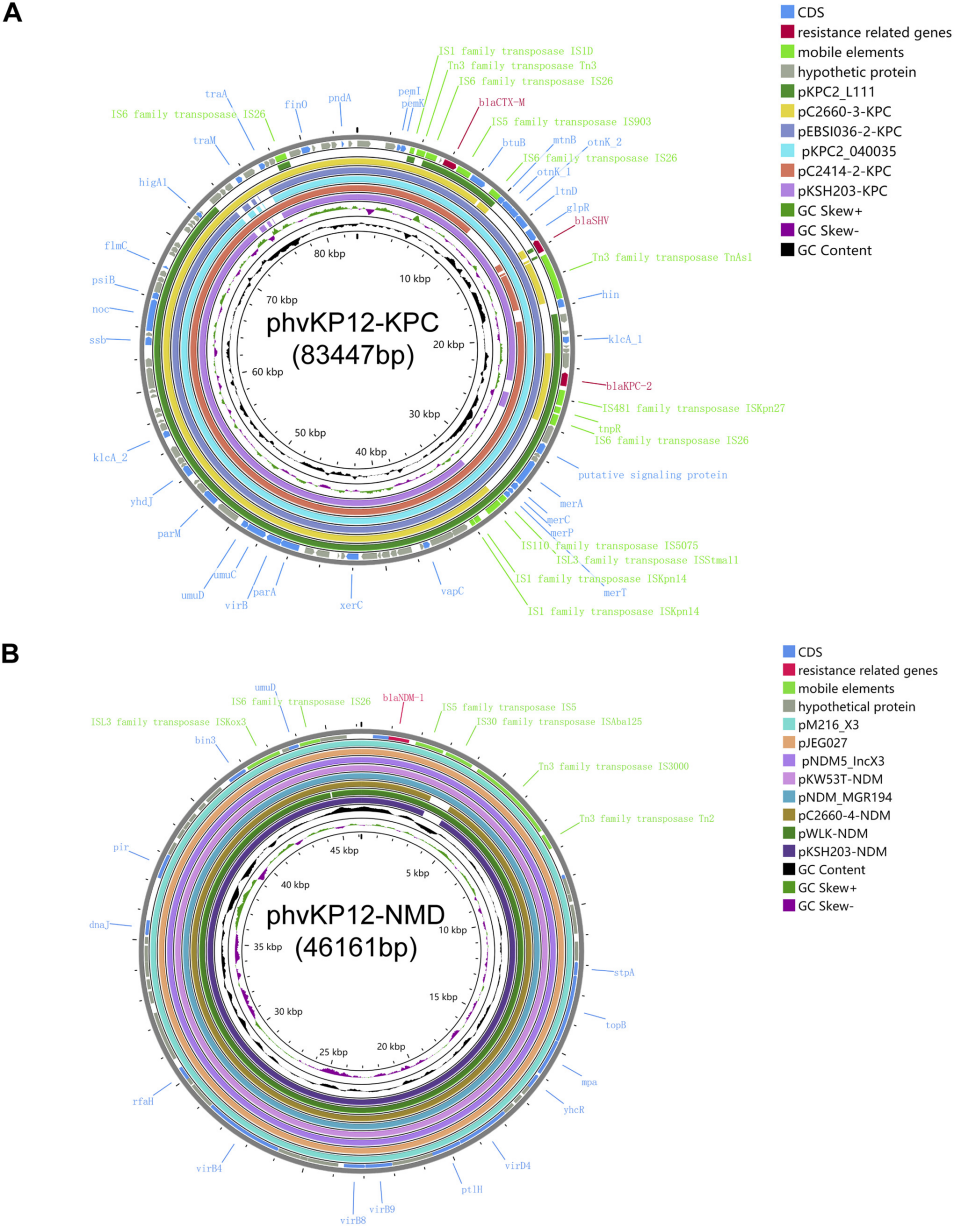
Circular map of the phvKP12-KPC and phvKP12-NDM plasmids.

**FIG 4:**
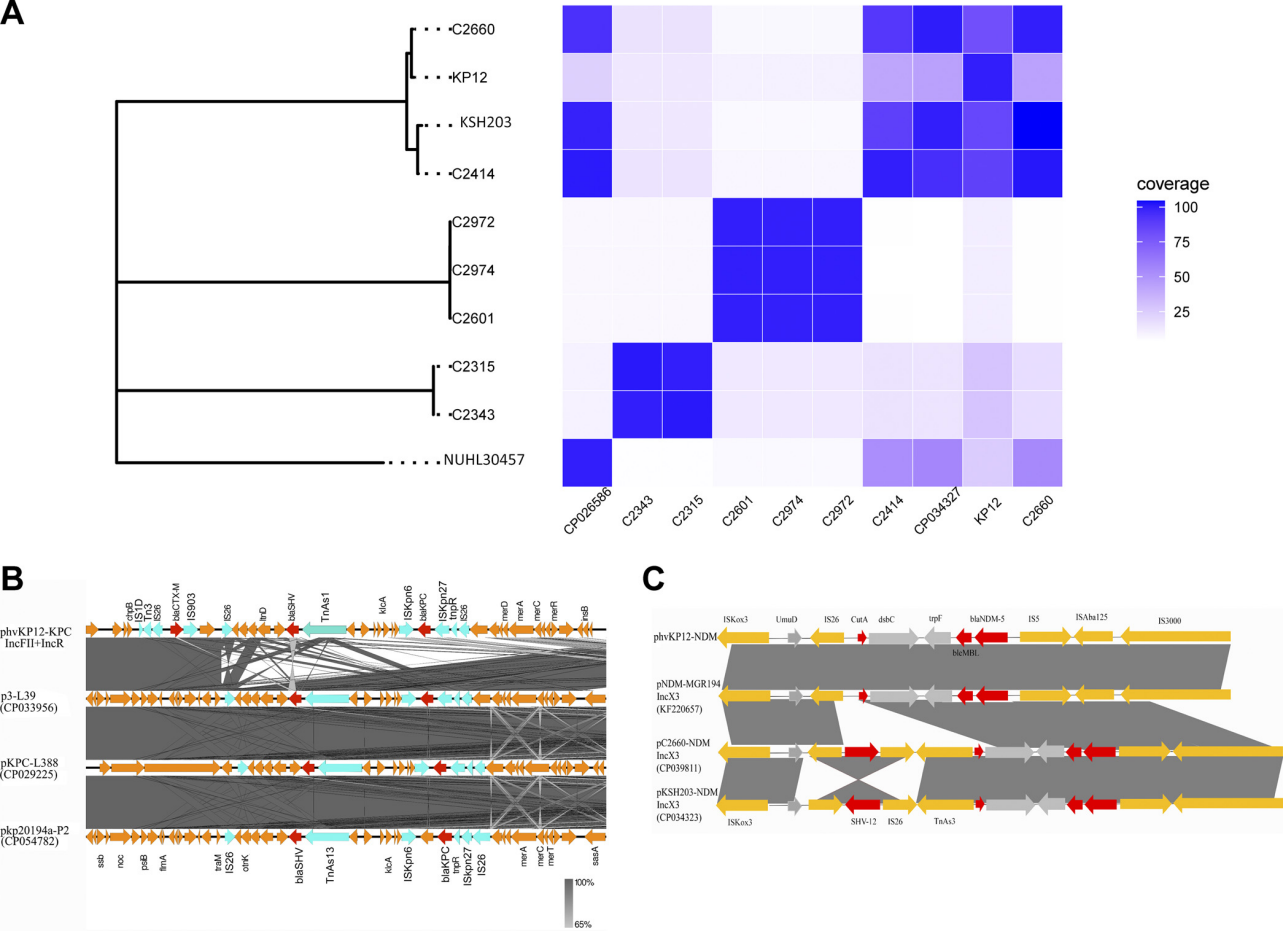
(A) Genome evolution and plasmid similarity analyses. (Left) Genome phylogenetic tree. (Right) similarity matrix of the KPC-2 and NDM-5 plasmids. The coverage of the homologous fragments is defined as the similarity between the query plasmid (row) and the subject plasmid (column). (B and C) Comparative analysis of similar plasmids.

Comparative analysis of the assembled plasmid sequences (Fig. 3A) revealed that the IncR:IncFII-type plasmid phvKP12-KPC shared 100% identity and 95 to 100% query coverage with the plasmids pKPC-CR-hvKP-C789 (GenBank accession number CP034417.1), pTZ40-KPC (accession number MT810372.1), pKPC2_040035 (accession number CP028796.1), pKPC2_L111 (accession number CP030134.1), and p3_L39 (accession number CP033956.1). The phvKP12-KPC plasmid contained resistance genes, including the carbapenemase-encoding gene *bla*_KPC-2_ as well as the two β-lactamase-encoding genes *bla*_CTX-M-65_ and *bla*_SHV-12_. The *bla*_KPC-2_ and *bla*_SHV-12_ genes were frequently found next to one another and were separated by Tn*As1*. This segment, carrying *bla*_KPC-2_ and *bla*_SHV-12_ of the phvKP12-KPC plasmid, was highly homologous to the pKPC-L388 plasmid (100% query coverage and 100% identity), p3-L39 (96% query coverage and 99.99% identity), and KP20194a-p2 (96% query coverage and 99.99% identity). Besides, another resistance region carried the *bla*_CTX-M-65_ gene, which contained a Tn*3*-IS*26*-*bla*_CTX-M-65_-IS*903*-*butB*-IS*26* structure and was similar to pEBSI036-2-KPC (GenBank accession number MT648513) and pKPC2_040035 (accession number CP028796.1). In most *Enterobacteriaceae* and Pseudomonas species, *bla*_KPC-2_ appears to be associated with a Tn*4401*-like transposon (Tn*3*-IS*Kpn27*-*bla*_KPC-2_-IS*Kpn6*) carried by different plasmids (17). However, the *bla*_KPC-2_ genetic environment consisting of IS*26*-*tnpR*-IS*Kpn27*-*bla*_KPC-2_-IS*Kpn6*-ORF (open reading frame)-*klcA*-Tn*As1*-*bla*_SHV-12_-IS*26* was found in the phvKP12-KPC plasmid (Fig. 4B). This structure illustrated that *bla*_KPC-2_ transposition was mediated by IS*26* instead the normal Tn*4401*.

The phVKP12-NDM plasmid was an IncX3-type plasmid of 46,161 bp. The plasmid structural region and the multiple-drug resistance regions were shown by plasmid comparison circle maps (Fig. 3B) and a horizontal gene environment map (Fig. 4C). BLAST analysis revealed that the phVKP12-NDM plasmid was 100% identical to pNDM5_IncX3 (GenBank accession number KU761328.1), pNDM-HN308 (accession number JX104760.1), and pNDM-MGR194 (accession number KF220657). Moreover, it shared 99% identity with pC2660-4-NDM (accession number CP039811) and pKSH203-NDM (accession number CP034323). The NDM-5 gene was flanked in the upstream region by IS*3000*-IS*5*-IS*Aba125*-*bla*_NDM-5_-*ble*_MBL_-*trpF*-*tat*-*dsbC*-IS*26*-*umuD*-IS*Kox3*, and this genetic background was identical to the pNDM_MGR194 from *Klebsiella pneumoniae* from India, pC2660-4-NDM from *Klebsiella pneumoniae* from China, and pKSH203-NDM (accession number CP034323) from *Klebsiella pneumoniae* from China. This genetic environment was identical to those found in various *bla*_NDM-1_-carrying plasmids in *Enterobacteriaceae* in China, such as pNDM-5-1140 (accession number MH985166), an NDM plasmid designated type A-IncX3 (18). However, the *bla*_SHV-12_ multiple-resistance region was deleted compared with pC2660-NDM and pKSH203-NDM, two plasmids from clinical strains cocarrying *bla*_KPC-2_ and *bla*_NDM-1_ in China.

### Evolutionary pathway of KPC-NDM-producing carbapenem-resistant *Enterobacteriaceae* (KPC-NDM-CRE).

**FIG 5:**
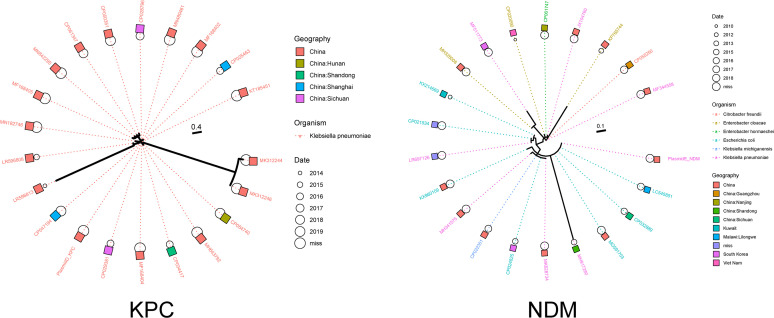
Comparison of the evolutionary characteristics of pKP12-KPC and pKP12-NDM. Plasmid labels are colored according to the organism, filled bars indicate the geographic location, and the sizes of the circles represent collection dates (the larger the circle, the more recent).

**FIG 6:**
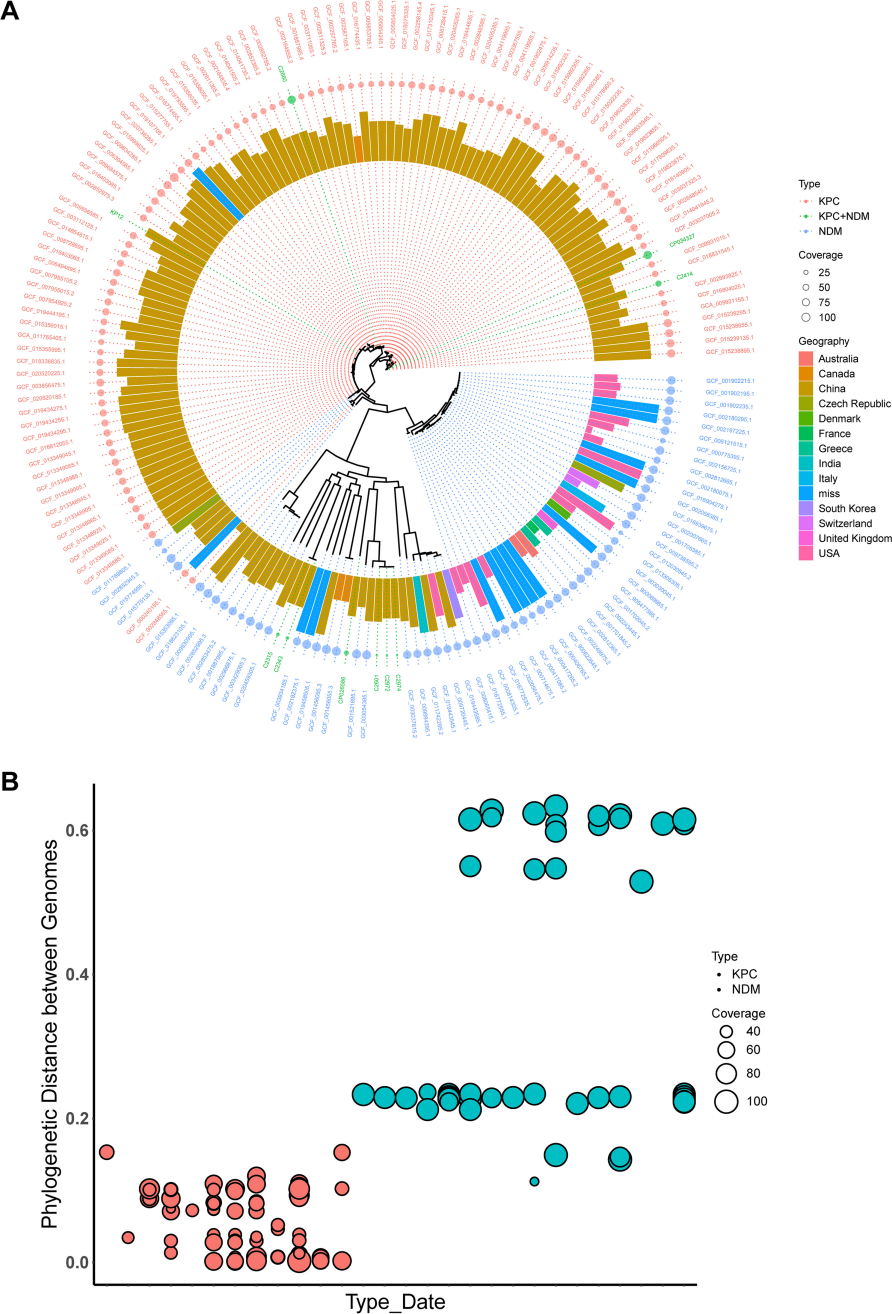
Evolutionary pathway inference for KPC-NDM-CRE. (A) Klebsiella pneumoniae genome phylogenetic tree. From inside to outside, following the Klebsiella pneumoniae genome phylogenetic tree, the height of the bar indicates the strain collection date, the color of the bar graph indicates the strain’s regional information, the size of the circle indicates the similarity to the hvKP12 *bla*_KPC_ and *bla*_NDM_ plasmids, and the color of the circle and outermost circle labels indicates the type of strain containing the plasmids. (B) Phylogenetic distance between genomes. The ordinate indicates the genetic distance between the subject genomes and the hvKP12 genome determined by phylogenetic analysis; the abscissa indicates the plasmid type and date of collection. Red circles indicate plasmids similar to *bla*_KPC_ plasmids, green circles indicate plasmids similar to *bla*_NDM_ plasmids, and the size of the circle indicates the similarity to the *bla*_KPC_ or *bla*_NDM_ plasmids.

The genomic phylogenetic tree showed that hvKP12 belonged to a single cluster group with C2660 (Fig. 4A). Additionally, we observed that the similarity between the KPC-2 and NDM-5 plasmids was consistent with genome evolution. Plasmid similarity analysis showed that the KPC-2 plasmid of hvKP12 had the highest similarity to those of strains C2660, KSH203 (accession number CP034327), and C2424. However, the NDM-5 plasmid was similar to those of strains C2660 and KSH203. Interestingly, the genetically similar KPC plasmids were limited to K. pneumoniae, but the NDM plasmids were similar to those of diverse host species (Fig. 5A). Moreover, similar KPC plasmids were found only in China, while similar NDM plasmids were discovered in different countries such as South Korea, Vietnam, Kuwait, and Malawi (Lilongwe). All publicly available data for K. pneumoniae strains bearing similar *bla*_KPC-2_ or *bla*_NDM-5_ plasmids were combined to evaluate the evolutionary process of hvKP12. According to Fig. 6B, the K. pneumoniae strains carrying plasmids resembling phvKP12-KPC had genomes closer to hvKP12, while the genomes of the strains carrying plasmids resembling phvKP12-NDM displayed a longer distance from hvKP12.

## DISCUSSION

Due to limited treatment choices, the rise of carbapenem-resistant clinical strains has become a severe clinical challenge. The occurrence of multiple carbapenemases in the same strain aggravates this situation (19). Not only did ST11-KL64 CRKP isolates cause numerous, periodic, distantly transmitted infections, but they also colonized the gastrointestinal tract, even when tigecycline and colistin were employed, providing the potential for hypervirulence (20). Previously reported strains cocarrying *bla*_KPC_ and *bla*_NDM_ were tigecycline-sensitive isolates, except for a recently reported ST464-type CR-hvKP strain named AHSWKP25 (21). However, the AHSWKP25 strain did not contain a typical hypervirulence plasmid and virulence genes like *iutA* or *rmpA*. Furthermore, ST464 is not common in China, while ST11 has a high prevalence (22). We also researched the evolutionary pathways of hvKP cocarrying *bla*_KPC_ and *bla*_NDM_ to explain the evolutionary mechanisms. In addition, other similar strains carrying both KPC and NDM have different resistance phenotypes, molecular phenotypes, and virulence phenotypes than hvKP12, as described in Table S1 in the supplemental material. This study reported an ST11 hvKP isolate cocarrying *bla*_KPC_ and *bla*_NDM_ with tigecycline resistance causing a fatal blood infection. According to the case records, the patient was first infected with a hypervirulent K. pneumoniae strain that was sensitive to almost all antibiotics. Next, it became resistant to carbapenems and tigecycline during antibiotic administration. We supposed that under such high-intensity antibiotic selection pressure, hvKP acquired resistance genes such as *bla*_KPC_ and *bla*_NDM_, as reported previously by Feng et al. (23). Long-term selective pressure by tigecycline induced gene mutations and increased the expression levels of efflux pumps, thereby reducing tigecycline insensitivity (24). Previously, it was found that mutations in the *tet*(A) genes were the primary mechanism of tigecycline resistance among CRKP isolates (16). In our study, *tet*(A) variants with type 1 mutations were also identified in the hvKP12 strain. However, we also found that hvKP12 had new mutations, including A93T, L169F, and F163T. Their specific effects on tigecycline resistance require further investigation. Conjugation experiments verified the ability of tigecycline resistance to be transferred by *tet*(A) point mutations (25). This may also be responsible for the increasing rate of tigecycline resistance. Different efflux pump genes, including *marA*, *ramA*, *rarA*, and *oqxB*, showed elevated expression levels in hvKP12. A recent study demonstrated that tigecycline resistance development in hvKP strains resulted in virulence deficiencies linked to decreased hypermucoviscosity (24). This evidence might explain the negative string assay but the presence of the *rmpA* and *rmpA2* genes in hvKP12. Accordingly, obsolescent hypermucoviscosity also deserves further exploration. As tigecycline was usually used to fight CRKP infections, this finding calls for therapeutic caution, and we must emphasize the evolutionary resistance of CRKP to tigecycline, or else an epidemic of tigecycline resistance may pose a fatal threat.

Using genomic cluster analysis techniques, we discovered that hvKP12 and C2660 were virtually identical on the genomic phylogenetic tree, indicating that the two KPC- and NDM-producing CRKP (KPC-NDM-CRKP) isolates may originate from the same ancestor. The difference between them is that the plasmids bearing resistance genes of C2660 can undergo interspecific transfer. However, the high virulence of hvKP12 posed far more harm than C2660. The common denominator was that both the KPC and NDM plasmids were very stable *in vivo*, which provided favorable conditions for the spread of the strains. The high degree of association between strains from different regions suggested that ST11-KL64 is a potentially epidemic type that deserves more attention. Next, whole-genome sequencing data of hvKP12 and bioinformatic analyses were used to further investigate how CRKP evolved into KPC-NDM-CRKP. Briefly, all publicly available data on K. pneumoniae strains harboring similar *bla*_KPC-2_ or *bla*_NDM-5_ plasmids in the NCBI database were analyzed to explore whether the genomes and plasmids evolved synchronously. As expected, our results are consistent with the previously proposed hypothesis that KPC-2–NDM-1–CRKP emerged from a KPC-2–CRKP progenitor, which acquired another highly transferable *bla*_NDM-1_ plasmid later (26). However, this hypothesis needs more genomic information to be verified. With the current high prevalence of KPC-2–CRKP, we need to be alert to the transfer of NDM plasmids to KPC-2–CRKP isolates, thus producing superresistant bacteria.

Gene environmental analysis indicated that the *bla*_KPC-2_ and *bla*_SHV-12_ genes were moved by the IS*26* transposable unit (TU), forming a new segment. According to Cai et al., this could be because IS*26* and the surrounding area comprised IS*Kpn27*-*bla*_KPC-2_-IS*Kpn6*-ORF-*klcA*-Tn*As1*-*bla*_SHV-12_, forming a TU (27). Specifically, the *bla*_NDM-5_ genetic background of hvKP12 was identical to that of the typical IncX3 plasmids. With a high prevalence in different geographic regions and species, IncX3 plasmids contains different *bla*_NDM_ alleles, including *bla*_NDM-1_, *bla*_NDM-4_, *bla*_NDM-5_, and *bla*_NDM-7_, which highlighted the role played by plasmids of this replicon type in the global dissemination of NDM-type carbapenem (28). The high prevalence of the IncX3-type NDM plasmid reminds us to strengthen its monitoring and increase caution against the strong likelihood of *bla*_NDM_ plasmids integrating into local *Enterobacteriaceae* strains, extending their resistance phenotype (18).

In conclusion, this is the first report of an ST11-KL64 CR-hvKP strain with tigecycline resistance coproducing *bla*_KPC_ and *bla*_NDM_ that caused a fatal blood infection. Interestingly, *bla*_KPC-2_ was located in a new segment mediated by the mobile element IS*26*. The evolutionary pathway of hvKP12 suggested that the KPC-2–CRKP strain acquired the *bla*_NDM-5_ plasmid and subsequently evolved into a KPC-NDM-CRKP strain. As ST11-KL64 CRKP strains have transmitted broadly throughout the world, the importance of infection control and the prevention of potential outbreaks of KPC-NDM-CRKP infections should be highlighted.

## MATERIALS AND METHODS

### Bacterial isolates and clinical information.

We obtained K. pneumoniae from a blood sample of a 74-year-old female in the First Affiliated Hospital of Chongqing Medical University (Chongqing, China), which was named hvKP12. Vitek 2 Compact and 16s DNA sequencing were performed to confirm this isolate. The *bla*_KPC_ and *bla*_NDM_ genes were detected by PCR and Sanger sequencing (29). A standard and predetermined case report form was used to collect clinical information. For primers and medical records see Table S2 and Table S3.

### Antimicrobial susceptibility and string testing.

Susceptibility to antimicrobial agents was assessed using the broth microdilution method according to Clinical and Laboratory Standards Institute (CLSI) guidelines (30). Tigecycline and colistin MICs were interpreted according to EUCAST guidelines (available at https://www.eucast.org/). Escherichia coli ATCC 25922 was used as a quality control. The hypermucoviscous phenotype of K. pneumoniae was confirmed using the string test (31).

### Conjugation experiments and transconjugant characteristics.

A conjugation test was carried out using hvKP12 as the donor. E. coli 600 with rifampin resistance was the recipient. Transconjugants were selected on Mueller-Hinton agar (MHA; Oxoid) plates containing 200 mg/L rifampin and 2 mg/L meropenem. Antimicrobial susceptibility testing (AST) and PCR amplification of the transconjugants were then performed to demonstrate the successful transfer of the plasmid to the recipient.

### Tigecycline resistance mechanism.

The tigecycline resistance genes *ramR*, *acrR*, *rpsJ*, *oqxR*, *tet*(A), *tet*(X), and *tet*(M) were detected by PCR and Sanger sequencing using primers reported previously (16). For mutation analysis, we compared the sequences of each gene to those of wild-type reference sequences, E. coli plasmid RP1 (GenBank accession number X00006) for the *tet*(A), *tet*(X), and *tet*(M) genes and K. pneumoniae MGH78578 (accession number CP000647) for the remaining genes. A quantitative Real-time PCR (qPCR) assay was used to assess the expression levels of the efflux pump genes (*acrA*, *acrB*, and *oqxB*) and their regulator genes (*ramA*, *marA*, *rarA*, and *soxS*) (32). The qPCR experiments were carried out in triplicate, and expression levels were calculated using the 2^−ΔΔ^*^CT^* method.

### Whole-genome sequencing and analysis.

Whole-genome sequencing (WGS) was performed on hvKP12. To summarize, complete genomic DNA was extracted using the Wizard genomic DNA purification kit (Promega) and sequenced using the PacBio RS II Single Molecule Real Time (SMAT) (Pacific Biosciences, CA) and Illumina X Ten (Illumina, San Diego, CA) sequencing platforms. Quality shearing of the Illumina sequencing data by Trimmomatic version 0.36 (http://www.usadellab.org/cms/?page=trimmomatic) yielded relatively accurate and valid data. Sequence correction was performed using PrInSeS-G version 1.1.0 (https://updeplasrv1.epfl.ch/prinses/) to correct editing errors and insertions-deletions of small fragments during splicing. Furthermore, contigs were complemented using GAPFiller version 1.11 (https://www.baseclear.com/genomics/bioinformatics/). Spliced second-generation sequencing data were assembled using SPAdes version 3.5.0 (http://cab.spbu.ru/software/spades/). Genes were predicted using Prokka version 1.10 (https://github.com/tseemann/prokka). Antibiotic resistance genes were predicted by ResFinder version 4.1 (https://cge.food.dtu.dk/services/ResFinder/). Prediction of the virulence genes was performed using VFDB (http://www.mgc.ac.cn/VFs/main.htm). Multilocus sequence typing (MLST) results and plasmid incompatibility types were analyzed using MLST version 2.0 (https://bitbucket.org/genomicepidemiology/mlst/src/master/) and PlasmidFinder version 2.1 (https://bitbucket.org/genomicepidemiology/plasmidfinder/src/master/). A complete K. pneumoniae K-locus reference database is available at https://github.com/katholt/Kaptive. Plasmid sequence comparisons and map generation were performed using the BLASTn algorithm on the cgview online server (http://cgview.ca/) (33). Easyfig (http://mjsull.github.io/Easyfig/) was used to compare the different mobile genetic elements.

### Phylogenic analysis.

Complete genomic data for hvKP12 with two plasmids, including both KPC and NDM, were obtained from PubMed. Genome evolution and plasmid similarity were studied with similarity matrices (ggtree). The phylogenetic tree was built based on similar KPC and NDM plasmid sequences uploaded to the NCBI database. The inclusion criteria were an identity of >90%, a query coverage of >90%, and a length of >2 kbp. Next, a phylogenetic tree was constructed using kSNP3 and ggtree. The evolutionary pathway was created using the distance between the phylogenetic tree and the coverage of the sequences, and the pathway was then visualized as a scatterplot in R.

### Ethics statement.

This study was approved by the Research Ethics Committee of the First Affiliated Hospital of Chongqing Medical University with a waiver of informed consent.

### Data availability.

All sequencing data have been deposited in the NCBI database under BioProject accession number PRJNA871048.
