# Supplementary material for: Emergence of a Fatal ST11-KL64 Tigecycline-Resistant Hypervirulent Klebsiella pneumoniae Clone Cocarrying blaNDM and blaKPC in Plasmids
Source: Microbiol Spectr. 2022 Oct 7;10(6):e02539-22. doi: 10.1128/spectrum.02539-22 (PMC9769963; doi:10.1128/spectrum.02539-22)
Supplement: Supplemental file 1 — Supplemental material. Download spectrum.02539-22-s0001.pdf, PDF file, 0.2 MB [file spectrum.02539-22-s0001.pdf]

Supplement Table S1. Reported information of co-existing KPC and NDM

Isolate	organism	sample	year	country	ST	serotype	Resistance genes	Tigecycline	NDM plasmid type	KPC plasmid type	reference
IR98	Klebsiella pneumoniae	Urine	2010	India	/	/	KPC-2,NDM-1	Sensitive	Conjugantable plasmid IncA/C	unconjugantable plasmid and non-type	[1]
WCHECl-14653	Enterobacter cloacae	Blood	2014	China	ST177	/	KPC-2,NDM-1	Sensitive	Conjugantable plasmid IncF	unconjugantable plasmid IncF	[2]
ADDIN EN.CITE CCBH14397	Enterobacter hosei	/	/	Brazil	ST15	/	KPC-2,NDM-1	Sensitive	Inc A/C	(IncN)	[3]
E14	Enterobacter cloacae	/	2013	Brazil	/	/	KPC-2,NDM-1	Sensitive	Conjugantable plasmid Inc/repP	chromosome	[4]
SZECL1	Enterobacter cloacae	/	2015	China	ST231	/	KPC-3,NDM-1	Sensitive	unconjugantable plasmid IncA/C2and IncR	Conjugantable plasmid IncX6	[5]
112298	Citrobacter freundii	/	2013	China	/	/	KPC-2,NDM-1	Sensitive	Conjugantable plasmid IncX3	unconjugantable plasmid and non-type	[6]
NUHL30457	HypervirulentKlebsiella pneumoniae	Wound secretions	2016	China	ST86	K2	KPC-2,NDM-1	Sensitive	IncFII(k)	IncN	[7]
HNTS79	Escherichia coli	vegetable	2015	China	TS79e	/	KPC-2,NDM-1	Sensitive	unconjugantable plasmid IncA/C	Conjugantable plasmid and non-type	[8]
WLK218	Raoultella	River sediment	2019	China	/	/	KPC-2,NDM-1	Sensitive	IncX3	non-type	[9]
C2315	Klebsiella pneumoniae	Ascites	2017	China	ST3493	K140	KPC-2,NDM-1	Sensitive	IncHI1B	IncFIA(HI1)+IncFI	[10]
C2343	Klebsiella pneumoniae	Ascites	2017	China	ST3493	K140	KPC-2,NDM-1	Sensitive	IncHI1B	IncFIA(HI1)+IncFI	[10]

C2414	Klebsiella pneumoniae	Urine	2017	China	ST11	KL47	KPC-2,NDM-1	Sensitive	Conjugantable plasmid IncN	IncR+IncFII	[10]
C2601	Klebsiella pneumoniae	Sputum	2017	China	ST15	K19	KPC-2,NDM-1	Sensitive	Conjugantable plasmid IncN2	IncR+IncFII	[10]
C2660	Klebsiella pneumoniae	Pus secretion	2017	China	ST11	KL64	KPC-2,NDM-1	Sensitive	Conjugantable plasmid IncX3	Conjugantable plasmid IncR+IncFII	[10]
C2972	Klebsiella pneumoniae	Sputum	2016	China	ST15	KL47	KPC-2,NDM-1	Sensitive	Conjugantable plasmid IncN2	Conjugantable plasmid IncFII	[10]
C2974	Klebsiella pneumoniae	Sputum	2016	China	ST15	KL47	KPC-2,NDM-1	Sensitive	Conjugantable plasmid IncN2	Conjugantable plasmid IncFII	[10]
KSH203	Klebsiella pneumoniae	Blood	2018	China	ST11	K25	KPC-2,NDM-1	Sensitive	IncX3	IncFII	[11]
KP1411	Klebsiella pneumoniae	Blood	2012	Brazil	ST340	/	KPC-2,NDM-1	Sensitive	/	/	[12]
KP1581	Klebsiella pneumoniae	Blood	2012	Brazil	ST340	/	KPC-2,NDM-1	Sensitive	/	/	[12]
KP4301	Klebsiella pneumoniae	Blood	2014	Brazil	ST340	/	KPC-2,NDM-1	Sensitive	/	/	[12]
SM1756	Serratia marcescens	Blood	2013	Brazil		/	KPC-2,NDM-1	Sensitive	/	/	[12]
KP4990	Klebsiella pneumoniae	Blood	2016	Brazil	ST258	/	KPC-2,NDM-1	Sensitive	/	/	[12]
EBSI036	HypervirulentKlebsiella pneumoniae	Blood	2019	Egypt	ST11	KL47	KPC-2,NDM-1	Sensitive	IncFIB:IncHI1B	ncR:IncFII	[13]
MH9CRKP	Klebsiella pneumoniae	Horse dung	2019	China	ST11	/	KPC-2,NDM-5	Sensitive	Conjugantable plasmid IncX3	unconjugantable plasmid F33:A-:B-	[14]

KPWX136	HypervirulentKlebsiella pneumoniae	Bronchial lavage fluid	2020	China	ST11	KL64	KPC-2,NDM-5	Sensitive	IncB/O/K/Z	IncFII	[15]
KOX3	Klebsiella oxytoca	Urine	2013	China	/	/	KPC-2,NDM-1，IMP-4	Sensitive	unconjugantable plasmid IncX3	unconjugantable plasmid IncP-6	[16]
AHSWKP25	HypervirulentKlebsiella pneumoniae	Blood	2021	China	ST464	KL725	KPC-2,NDM-1	Resistant	conjugantable plasmid IncX3	unconjugantable plasmid	[17]

Note: / indicates that the article is not mentioned

Supplement Table S2. The primers used to detect the resistance gene

Gene		Forward sequence (5′→3′)	Reverse sequence (5′→3′)	Reference
Carbapenems	*bla*KPC	CGTCTAGTTCTGCTGTCTTG	CTTGTCATCCTTGTTAGGCG	[18]
	*bla*NDM	GGTTTGGCGATCTGGTTTTC	CGGAATGGCTCATCACGATC	
	*bla*IMP	GGAATAGAGTGGCTTAAYTC	GGTTTAAYAAAACAACCACC	
	*bla*VIM	GATGGTGTTTGGTCGCATA	CGAATGCGCAGCACCAG	
	*bla*OXA-48-like	GCGTGGTTAAGGATGAACAC	CATCAAGTTCAACCCAACCG	
Tigecycline-resistance Mutation detection	*ramR*	CACGGTTCATATCCTGACCA	CCRTCGACCTTAAACACGTC	[19]
	acrR	GCTAAGCTGCCTGAGAGCAT	ATGCAAATGCCGGAGAATAC	
	rpsJ	ACAGCCGGTTCGATATGA	AGTAACGCGGTTTGCTTC	
	oqxR	GTCACCAGAAAATGATTAATGCGC	GCCTTTGCCCGTGAAATCAG	
	tet(A)	GCCTTTCCTTTGGGTTCTCT	TGTCCGACAAGTTGCATGAT	
	tet(X)	CCCGAAAATCGWTTTGACAATCCTG	GTTTCTTCAACTTSCGTGTCGGTAAC	[20]
	tmexC	TGGCGGGGATCGTGCTCAAGCGCAC	CAGCGTGCCCTTGCKCTCGATATCG	

quantitative real-time PCR	*ramA*	GATATCGCTCGCCATGC	CTGTGGTTCTCTTTGCGGTAG	[21]
	acrA	ATGTGACGATAAACCGGCTC	CTGGCAGTTCGGTGGTTATT	
	acrB	AAACTTCGCCACTACGTCATA	AGCTTAACGCCTCGATCAT	
	marA	TGTCTGAGCGCTCCGGTTACT	TTCTGCGCAATCTCCGTCA	
	soxS	TACCTGCAGCGGATGTTC	AAGGTTTGCTGCGAGACGTAG	
	rarA	GTTTGTTGACGAAGTGCA	GCCATCATTTCCAGGGTA	[22]
	oqxB	CGAAGAAAGACCTCCCTACCC	CGCCGCCAATGAGATACA	
	rpoB	CGCGTATGTCCGATCGAAA	GCGTCTCAAGGAAGCCATATTC	
16s NDA	16s NDA	AGAGTTTGATCCTGGCTCAG	GGTTACCTTGTTACGACTT	online

Supplement Table 3. Clinical characteristics of patients with hvKP12

Variables	Age	Gender	District	Ward	Underlying Conditions	Specimen type	Infection type	Invasive procedures	Therapeutic antimicrobial usage	Length of stay (days)	Admission date	Outcome
Basic information	74	Female	Beibei	ICU	Hypertension,rheumatoid arthritis	blood	Sepsis	Yes	LVX, MEM, CAZ/AVI, TGC	24	12/03/2021	Died

MIC														
MIC	Antibiotics[a]	AZT	CAZ	IPM	CRO	FEP	MEM	AMK	TGC	LEV	FOX	CST	TZP	SCF
	hvKP[b]	**≤4**	**≤0.25**	**≤0.25**	**≤0.25**	**≤0.25**	**≤0.25**	**≤0.25**	**≤0.25**	**≤0.25**	**≤4**	**≤0.25**	**≤4**	**≤8**
	CR-hvKP[c]	≥256	≥256	≥256	≥128	≥128	≥128	16	8	128	≥256	**≤0.25**	≥128	≥256

[a] AZT,aztreonam;CAZ,ceftazidime;IMP,imipenem;CRO,cefatriaxone; FEP, cefepime; MEM,meropenem;AMK,amikacin; TGC, tigecycline; LVX,levofloxacin; FOX,cefoxitin;

CST,colistin;TZP,piperacillin/tazobactam;SCF,cefoperazone/sulbactam; CAZ/AVI,Ceftazidime/avibactam, Numbers shown in bold were susceptible judged by CLSI /EUCAST breakpoints.

[b] This isolate collected in March 17th, 2021

[c] This isolate collected in April 2nd, 2021

Reference

1. Kumarasamy K, Kalyanasundaram A. Emergence of *Klebsiella pneumoniae* isolate co-producing NDM-1 with KPC-2 from India. J Antimicrob Chemother. 2012 Jan;67(1):243-4.
2. Wu W, Feng Y, Carattoli A, et al. Characterization of an *Enterobacter cloacae* Strain Producing both KPC and NDM Carbapenemases by Whole-Genome Sequencing. Antimicrob Agents Chemother. 2015 Oct;59(10):6625-8.
3. Pereira PS, Borghi M, Albano RM, et al. Coproduction of NDM-1 and KPC-2 in *Enterobacter hormaechei* from Brazil. Microb Drug Resist. 2015 Apr;21(2):234-6.
4. Quiles MG, Rocchetti TT, Fehlberg LC, et al. Unusual association of NDM-1 with KPC-2 and armA among Brazilian *Enterobacteriaceae isolates*. Braz J Med Biol Res. 2015 Feb;48(2):174-7.
5. Du H, Chen L, Chavda KD, et al. Genomic Characterization of *Enterobacter cloacae* Isolates from China That Coproduce KPC-3 and NDM-1 Carbapenemases. Antimicrob Agents Chemother. 2016 Apr;60(4):2519-23.
6. Feng J, Qiu Y, Yin Z, et al. Coexistence of a novel KPC-2-encoding MDR plasmid and an NDM-1-encoding pNDM-HN380-like plasmid in a clinical isolate of *Citrobacter freundii*. J Antimicrob Chemother. 2015 Nov;70(11):2987-91.
7. Liu Y, Long D, Xiang TX, et al. Whole genome assembly and functional portrait of hypervirulent extensively drug-resistant NDM-1 and KPC-2 co-producing *Klebsiella pneumoniae* of capsular serotype K2 and ST86. J Antimicrob Chemother. 2019 May 1;74(5):1233-1240.
8. Wang J, Yao X, Luo J, et al. Emergence of *Escherichia coli* co-producing NDM-1 and KPC-2 carbapenemases from a retail vegetable, China. J Antimicrob Chemother. 2018 Jan 1;73(1):252-254.
9. Dang B, Zhang H, Li Z, et al. Coexistence of the blaNDM-1-carrying plasmid pWLK-NDM and the blaKPC-2-carrying plasmid pWLK-KPC in a *Raoultella ornithinolytica* isolate. Sci Rep. 2020 Feb 11;10(1):2360.
10. Gao H, Liu Y, Wang R, et al. The transferability and evolution of NDM-1 and KPC-2 co-producing *Klebsiella pneumoniae* from clinical settings. EBioMedicine. 2020 Jan;51:102599.
11. Fu L, Wang S, Zhang Z, et al. Whole genome sequence of blaNDM and blaKPC co-producing *Klebsiella pneumoniae* isolate KSH203 with capsular serotype K25 belonging to ST11 from China. J Glob Antimicrob Resist. 2020 Mar;20:272-274.
12. Bes T, Nagano D, Martins R, et al. Bloodstream Infections caused by *Klebsiella pneumoniae* and Serratia marcescens isolates co-harboring NDM-1 and KPC-2. Ann Clin Microbiol Antimicrob. 2021 Aug 30;20(1):57.
13. Ahmed M, Yang Y, Yang Y, et al. Emergence of Hypervirulent Carbapenem-Resistant *Klebsiella pneumoniae* Coharboring a bla NDM-1-Carrying Virulent Plasmid and a bla KPC-2-Carrying Plasmid in an Egyptian Hospital. mSphere. 2021 May 19;6(3).
14. Wang H, Li X, Liu BT. Occurrence and characterization of KPC-2-producing ST11 *Klebsiella pneumoniae* isolate and NDM-5-producing Escherichia coli isolate from the same horse of equestrian clubs in China. Transbound Emerg Dis. 2021 Mar;68(2):224-232.
15. Hu R, Li Q, Zhang F, et al. Characterisation of blaNDM-5 and blaKPC-2 co-occurrence in K64-ST11 carbapenem-resistant *Klebsiella pneumoniae*. J Glob Antimicrob Resist. 2021 Sep 2;27:63-66.
16. Wang J. First Report of Klebsiella oxytoca Strain Simultaneously Producing NDM-1, IMP-4, and KPC-2 Carbapenemases. Antimicrob Agents Chemother. 2017.
17. Hao J, Zhang B, Deng J, et al. Emergence of a Hypervirulent Tigecycline-Resistant *Klebsiella pneumoniae* Strain Co-producing bla NDM-1 and bla KPC-2 With an Uncommon Sequence Type ST464 in Southwestern China. Front Microbiol. 2022;13:868705.
18. Poirel L, Walsh TR, Cuvillier V, et al. Multiplex PCR for detection of acquired carbapenemase genes. Diagn Microbiol Infect Dis. 2011 May;70(1):119-23.

19. Chiu SK, Huang LY, Chen H, et al. Roles of ramR and tet(A) Mutations in Conferring Tigecycline Resistance in Carbapenem-Resistant *Klebsiella pneumoniae* Clinical Isolates. Antimicrob Agents Chemother. 2017 Aug;61(8).

20. Hirabayashi A, Dao TD, Takemura T, et al. A Transferable IncC-IncX3 Hybrid Plasmid Cocarrying blaNDM-4, tet(X), and tmexCD3-toprJ3 Confers Resistance to Carbapenem and Tigecycline. mSphere. 2021 Aug 25;6(4):e0059221.

21. Wang X, Chen H, Zhang Y, et al. Genetic characterisation of clinical *Klebsiella pneumoniae* isolates with reduced susceptibility to tigecycline: Role of the global regulator RamA and its local repressor RamR. Int J Antimicrob Agents. 2015 Jun;45(6):635-40.

22. He F, Fu Y, Chen Q, et al. Tigecycline susceptibility and the role of efflux pumps in tigecycline resistance in KPC-producing *Klebsiella pneumoniae*. PLoS One. 2015;10(3):e0119064.
